# A98 EFFECT OF A PROLONGED MULTIVITMAIN SHORTAGE ON HOME PARENTERAL NUTRITION PATIENTS: SINGLE CENTRE EXPERIENCE

**DOI:** 10.1093/jcag/gwad061.098

**Published:** 2024-02-14

**Authors:** N C Lam, C Wang, K Schwenger, C Arca-Juico, K Chin, A Macgillivray, C Yuen, I Pang, J Allard

**Affiliations:** Medicine, Toronto General Hospital, Toronto, ON, Canada; Medicine, Toronto General Hospital, Toronto, ON, Canada; Medicine, Toronto General Hospital, Toronto, ON, Canada; Medicine, Toronto General Hospital, Toronto, ON, Canada; Medicine, Toronto General Hospital, Toronto, ON, Canada; Medicine, Toronto General Hospital, Toronto, ON, Canada; Medicine, Toronto General Hospital, Toronto, ON, Canada; Medicine, Toronto General Hospital, Toronto, ON, Canada; Medicine, Toronto General Hospital, Toronto, ON, Canada

## Abstract

**Background:**

Multivitamin shortages in home parenteral nutrition (HPN) are scarce but have a significant impact on patient morbidity. Recently, Ontario, Canada experienced a HPN intravenous multivitamin shortage that lasted over 3 months. As per the American Society of Parenteral and Enteral Nutrition (ASPEN) guidelines, patients were instructed to use an over-the-counter multivitamin supplement during the shortage. However, due to the nature of HPN patients, the risk for malnutrition related complications remains high, specifically thiamine deficiency.

**Aims:**

This study aims to assess the relationship between the multivitamin shortage and thiamine deficiency. We conducted an analysis before and after the intravenous multivitamin shortage in HPN patients and compared anthropometric, biochemical and clinical outcomes in those who followed the oral multivitamin recommendation versus those who did not.

**Methods:**

This retrospective descriptive study included HPN patients in a single-centre program in Toronto, Canada who required HPN ≥ 5 days/week. These patients were followed throughout the multivitamin shortage which lasted from February - June 2023. Patients were categorized into two groups: those who consumed an oral multivitamin and those who did not. Clinical and biochemical data were collected pre and post-multivitamin shortage. Data was collected on compliance with taking oral multivitamins. The primary outcome was the presence of any adverse clinical outcome related to a micronutrient deficiency.

**Results:**

Twenty-five HPN patients were included. 56% (n=14) of patients were compliant with oral multivitamin usage and met the ASPEN recommendations for supplementation. Compliant patients were significantly older and were on significantly longer duration of HPN. Two patients were diagnosed with WE and had brief hospitalizations. Both patients experienced complete resolution of their symptoms after treatment with high-dose thiamine. Hence, compliance with oral multivitamins did not correlate to developing WE. The median time from the shortage to the development of WE was 2 months. Though not statistically significant, those who developed WE were more likely to be female, utilized a venting gastrostomy tube, and utilized HPN for longer.

**Conclusions:**

Complications related to micronutrient deficiency are rare in the HPN population but shortages in intravenous multivitamins can increase the risk of conditions such as WE. In our study, compliance with oral multivitamins did not guarantee protection from developing adverse outcomes, likely due to limitations in absorption due to altered gastrointestinal anatomy. A regimented protocol for screening should be implemented in HPN patients, especially during long shortages of key HPN components like multivitamins.

**Funding Agencies:**

None

